# Effect of Different Ratios of Glycerol and Erythritol on Properties of Corn Starch-Based Films

**DOI:** 10.3389/fnut.2022.882682

**Published:** 2022-04-25

**Authors:** Bin Wang, Xin Xu, Youxin Fang, Shouxin Yan, Bo Cui, A. M. Abd El-Aty

**Affiliations:** ^1^State Key Laboratory of Biobased Material and Green Papermaking, Qilu University of Technology, Shandong Academy of Sciences, Jinan, China; ^2^School of Food Science and Engineering, Qilu University of Technology, Shandong Academy of Sciences, Jinan, China; ^3^Department of Food Science and Engineering, Shandong Agricultural University, Taian, China; ^4^Department of Forestry College, Shandong Agricultural University, Taian, China; ^5^Department of Pharmacology, Faculty of Veterinary Medicine, Cairo University, Giza, Egypt; ^6^Department of Medical Pharmacology, Faculty of Medicine, Atatürk University, Erzurum, Turkey

**Keywords:** corn starch-based films, glycerol, erythritol, physicochemical properties, plasticizer, bio-compatible plastics

## Abstract

The demand for biodegradable products has increased; hence, a suitable method for producing green composites is essential. This study prepared corn starch-based films using the solution casting method, and the physicochemical properties of the prepared films were investigated using a mixture of glycerol (GLY) and erythritol (ERY) at different ratios (4:0, 3:1, 2:2, 1:3, and 0:4) as plasticizing agents. The crystallinity, hydrophilicity, mechanical properties, oxygen and water vapor, surface roughness, and thermal stability of corn starch-based films were analyzed using small-angle X-ray diffraction, water contact angle, automatic tensile testing machine, oxygen permeability tester and water vapor permeability analyzer, atomic force microscope, and thermogravimetric analyzer. With the increase in GLY ratio, the thickness, water-solubility, water content, water vapor permeability, elongation at break, oxygen permeability and V-shaped crystallization of the corn starch-based films increased. The tensile strength and the thermal stability decreased with increasing the GLY ratio. We developed a new plasticizer using glycerol and erythritol to improve the properties of starch films and provided the basis for the industrial production of corn starch-based films.

## Introduction

Plastic pollution is a serious problem worldwide, which occurs during the entire treatment cycle of plastics, from chemical fuel production to incineration (1). At the end of the 20th century, approximately 260 billion bags of plastic were produced globally, with an industrial output value of US$ 1 trillion (2). Due to population growth, consumer demand for plastic products increases (3). As plastic production requires oil as a raw material, which is a non-renewable resource, the price of plastics will increase (4). In addition, environmental pollution caused by the production, use, and destruction of plastic materials is a serious concern as they contribute to global warming through CO_2_ production (5). Therefore, it is crucial to develop a suitable method for producing green composites. Starch-based films have become the focus of current research because of starch’s renewable and biodegradable characteristics. Starch films are mainly used in food packaging materials (6), as they have a low cost of production. They are considered to be an attractive choice in terms of economic and sustainable development (7).

Starch is one of the main carbohydrates and a vital energy source for humans and is widely used in the food industry (8). In addition, starch can be used as the main component of biodegradable materials (8). Starch is mainly composed of amylose and amylopectin. Amylose is a linear chain of glucose units connected by α-l,4 glycosidic linkages. In contrast, amylopectin is a short, highly branched macromolecule composed of d-glucopyranose units, connected by α-l,6 linkages (6). Amylopectin is the main component of the starch crystalline region, and the branching points of amylose and amylopectin are the main components of the starch amorphous region (9). It produces a tough gel, while when amylopectin is dispersed in water, it is more stable and produces a relatively soft gel.

Starch films have some limitations, such as poor mechanical properties, poor hydrophobicity, unsuitability to be converted into a film. Therefore, plasticizers must be added to optimize their performance. The primary function of plasticizers is to break the hydrogen bonds scattered between the polymer chains, separate the chains, and increase the starch films’ flexibility (6). Polyols (polyalcohols), including monosaccharides (such as glycerol, erythritol, xylitol, sorbitol, and mannitol) and disaccharides (maltitol and lactitol)-based polyols are low molecular weight carbohydrates used as a sweetener in food, non-food, health care, and pharmaceutical applications (10). Moreover, they are distributed between polymer chains to achieve plasticization (6). Erythritol is the first polyol produced by an entirely biotechnological process (11). Phase separation and crystallization of polyols, such as xylitol or sorbitol, when used as a single plasticizer at high concentrations in starch-based edible film (12). Both erythritol and glycerol have a polyhydroxy structure; however, erythritol is easily crystallized. Therefore, its hygroscopicity and water absorption are weaker than glycerol, which is not easily crystallized. In another word, glycerol has a higher hygroscopicity and water absorption than erythritol. In our preliminary study, we have added different concentrations of erythritol (0, 30, 35, 40, and 45%) to corn starch-based films, and we found starch films without erythritol cannot form a complete structure. The addition of erythritol can promote the formation of a complete starch films structure. With the increase of erythritol concentration, the elongation at the break of starch films increased, and the tensile strength decreased. Therefore, we assume that erythritol can be used as a starch films plasticizer, although the stability of erythritol plasticized starch films decreased due to crystallization of the erythritol (13). The high tendency to crystallize was the reason why erythritol was not used as a plasticizer in the films (10). As the type and dosage of plasticizers can affect starch films’ physical and chemical properties (6), we have conducted this study to investigate the physicochemical properties of the prepared films using a mixture of glycerol (GLY) and erythritol (ERY) at different ratios (4:0, 3:1, 2:2, 1:3, and 0:4) to prove our hypothesis.

## Materials and Methods

### Materials

Corn starch (amylose content: 33.81%, moisture content: 10.32%) was provided by the Zhucheng Xingmao corn development company (Zhucheng, China). GLY (Molecular weight: 92.09 Da, purity: ≥99.0%) and ERY (Molecular weight: 122.12 Da, Purity ≥ 98%) were supplied by Solebo (Beijing, China).

### Corn Starch-Based Film Preparation

**FIGURE 1:**
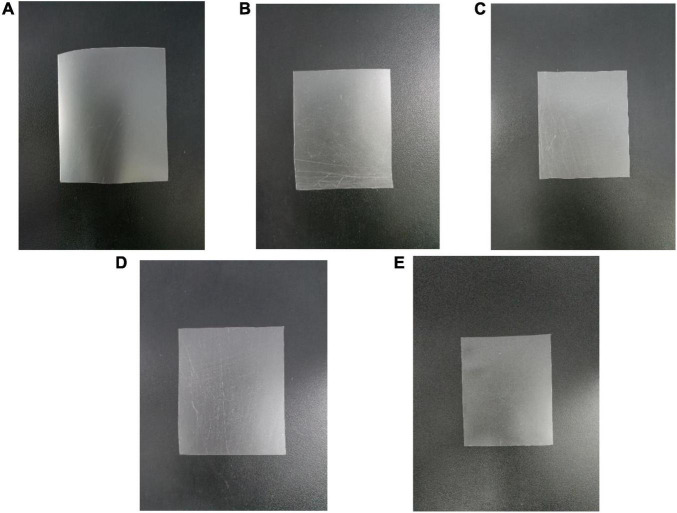
Pictorial presentation of corn starch-based films supplemented with GLY and ERY **(A)** (0:4), **(B)** (1:3), **(C)** (2:1), **(D)** (3:1), and **(E)** (4:0).

Corn starch-based films were prepared using the solution casting method (14). Briefly, 6 g of corn starch was weighed and dissolved in 100 mL of distilled water in a 250 mL conical flask. The conical flask was placed at 90°C in a constant-temperature water bath (B101S KeTai Company, Shanghai, China) with a rotational speed of 800 rpm for 30 min until the corn starch was fully gelatinized. After gelatinization, different ratios of GLY and ERY (4:0, 3:1, 1:1, 1:3, and 0:4) were added to the flask so that the total amount of the plasticizer was 30% (1.8 g) of the dry weight of corn starch. Then, the flask was placed in a constant-temperature water bath and stirred for 10 min at 90°C. After stirring, the film-forming solution was poured into a special polytetrafluoroethylene mold (PTFE, 15 cm × 20 cm, Shanghai Yuanye Co., Ltd., Shanghai, China), and the mold was dried in an oven at 45°C for 10 h. The corn starch-based films were then removed from the mold and balanced at 25°C and relative humidity (RH) of 53% for 48 h, until further use. The morphological property of corn starch-based films is shown in Figure 1.

### Thickness

After the corn starch-based films were balanced for 48 h, the thickness of the films was measured (15) using an electronic spiral micrometer (Mitutoyo No. 293-240-30, Tokyo, Japan) with an accuracy of 0.001 mm. Ten points were randomly selected on the corn starch-based films, and the average value was calculated.

### Water Solubility

The solubility of corn starch-based films was determined according to the method described by Wang et al. (16) with slight modifications. First, corn starch-based films were cut into square samples of 2 × 2 cm and weighed (M_1_). Next, the corn starch sample and 100 mL distilled water were added to a conical bottle, the bottle was placed at 25°C and stirred at 180 rpm for 6 h, and the sample was removed and dried in an oven at 110°C for 7 h. The samples were weighed (M_2_), and the solubility of the different corn starch-based films was calculated according to the following formula:


$$S=\frac{M_{1}-M_{2}}{M_{1}}\times 100$$

The experiment was repeated three times, and the average value was calculated.

### Moisture Content

After corn starch-based films were balanced for 48 h, they were cut into square pieces of 2 × 2 cm, and their mass was weighed (M_3_). Then, the corn starch-based film samples were placed in an oven at 110°C and dried for 24 h. After the drying was completed, the samples were removed, and each starch film sample was weighed (M_4_). The moisture content of the different corn starch-based films was calculated using the following formula:


$$M\,C=\frac{M_{3}-M_{4}}{M_{3}}\times 100$$

The experiment was repeated three times, and the average value was calculated.

### Water Contact Angle

A drop of water (volume 3 μL) was placed on the surface of starch-based films with the size of 5 cm × 5 cm. The surfaces should be smooth and wrinkle-free. As the drop of water spreads, it forms a dome shape across the surface. The contact interface between the droplet and the corn starch-based films was photographed when the droplet contacted the starch film for 10 s.

### Water Vapor Permeability (WVP)

Herein, we used W3, a 030 WVP tester (Lab think Instruments Co., Ltd., Jinan, China) (16). After balancing corn starch-based films for 48 h, they were cut into circular pieces with an area of 33 cm^2^. Circular corn starch-based film samples were then placed into the WVP tester. The time was set to 12 h, with a determination interval of 30 min. The average WVP of the corn starch-based films samples was obtained after measuring WVP of each sample three times.

### Oxygen Permeability (OP)

The gas permeability tester (Labthink Instruments Co., Ltd., Jinan, China) was used to measure the oxygen permeability of corn starch-based films. The parameters were 25°C, 53% relative humidity, and the tested area was 50 cm^2^. The corn starch-based films should be smooth and intact without scratches.

### Mechanical Properties

After the corn starch-based films were balanced, they were cut into rectangular pieces (1 × 10 cm), then placed on a tensile testing machine (Param Xlw Co., Ltd., Jinan, China), with a crosshead speed of 50 mm/min. At the end of the experiment, the tensile strength (TS, MPa) and elongation at break (EAB, %) were measured using an automatic tensile testing machine. The mechanical properties of each group of samples were measured at least five times, and the average value was used.

### X-Ray Diffraction (XRD)

After the corn starch-based films were balanced at 25°C and 50% RH for 48 h, each sample was installed on a special measuring glass sheet, and the crystallinity was measured using an X-ray diffractometer (Tokyo, Japan) (17, 18). The parameters of the X-ray diffractometer were as follows: Cukα radiation (λ = 1.54 A, 40 kV, and 30 mA), scanning range (2 θ) from 4° to 40°, and step size 3 °/min.

### Thermogravimetric Analysis (TGA)

A thermogravimetric analyzer was used to determine the thermal stability of the corn starch-based films (19, 20) and the relationship between the quality of the corn starch-based films and temperature. A 5 mg sample of corn starch-based films was weighed in a ceramic crucible. The heating program of the thermogravimetric analyzer was conducted over the temperature range of 30 – 600 °C, at a heating rate of 20°C/min. A thermodynamic degradation curve was drawn, and the TGA curve was obtained using derivative thermogravimetric analysis (DTG). The relationship between the corn starch-based films’ degradation rate and the temperature was determined using TGA and DTG curves.

### AFM

Atomic force microscopy can evaluate the surface roughness of corn starch-based films. The atomic force microscopy tapping mode was set at a scan size of 10 μm × 10 μm. After testing, NanoScopeAnalysis1.5 analysis software was used to analyze AFM data (Rq: Root mean square average of height deviation) (21).

### Statistical Analyses

Each group of experiments was repeated at least three times, and one-way analysis of variance was performed. Data were analyzed using SPSS software (version 13.0, Statistical Package for the Social Sciences Inc., Chicago, IL, United States). The difference between the average values of the data was compared using Duncan’s range test, and P < 0.05 was considered statistically significant.

## Results and Discussion

### Thickness

We observed that the thickness of the starch films increased with the increasing plasticizer ratio. The molecular basis for this may be that the plasticizer can penetrate the structural network of the corn starch-based films, resulting in a greater film thickness (22). As shown in Table 1, the thickness of corn starch-based films increases with the increases in GLY ratio, indicating that the effect of GLY on corn starch-based films thickness was greater than that of ERY (160 ± 3.47–138 ± 7.84 μm). This might be because GLY has strong hygroscopicity, and GLY absorbs more water than ERY under the same conditions, thus destroying and reorganizing the polymer chain network. Because plasticizers can destroy the intermolecular polymer chain network and produce more free volume. In turn, incorporating a plasticizer increased the thickness of the starch-based films (23).

**TABLE 1:** Functional properties of the corn starch-based films supplemented with GLY and ERY (4:0, 3:1, 2:2, 1:3, 0:4).

Sample	Thickness (μ m)	Moisture content (MC) (%)	Solubility in water (%)
GLY: ERY (4:0)	160 ± 3.47^a^	19.37 ± 1.75^a^	36.56 ± 3.58^a^
GLY: ERY (3:1)	155 ± 4.67^a^	15.50 ± 2.42^ab^	33.07 ± 4.62^b^
GLY: ERY (2:2)	153 ± 1.50^a^	13.95 ± 1.08^b^	30.77 ± 2.93^c^
GLY: ERY (1:3)	145 ± 8.70^ab^	13.68 ± 3.45^b^	27.79 ± 1.76^d^
GLY: ERY (0:4)	138 ± 7.84^b^	13.54 ± 5.03^b^	25.04 ± 1.81^e^

*Different superscript letters in each column showed a significant difference (P < 0.05).*

### Moisture Content

Moisture content is an important index of materials used to make starch films. Water molecules can play a plasticizing role in starch films because starch is a hydrophilic polymer (24). As shown in Table 1, the moisture content of corn starch-based films increased with an increase in GLY ratio because the plasticizer contains a higher amount of hydroxyl groups (25). In addition, as GLY is hygroscopic and retains huge volumes of water, its molecular structure is more similar to that of glucose than ERY. With an increase in ERY content, the molecular interaction between ERY and the starch polymer chain becomes stronger, weakening the interaction between ERY and water (26, 27). Therefore, with the increasing ERY ratio, the moisture content of the corn starch-based films decreased continuously.

### Mechanical Properties

**FIGURE 2:**
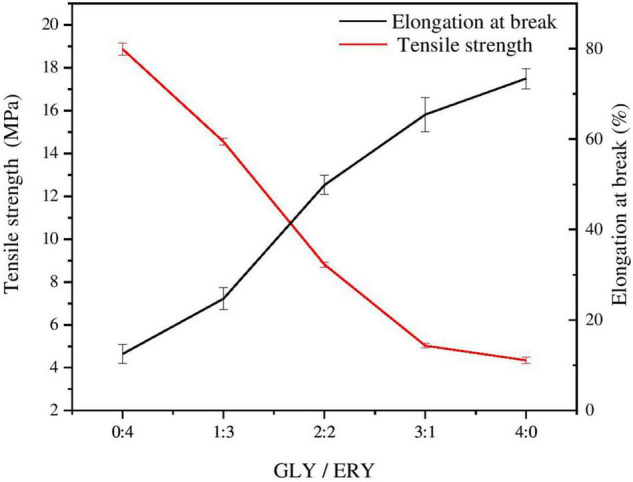
Tensile strength and elongation at break of corn starch-based films supplemented with GLY and ERY (0:4, 1:3, 2:1, 3:1, and 4:0).

The mechanical properties of corn starch-based films determine their use in the packaging industry, so the TS and EAB of corn starch-based films are important indicators for determining whether corn starch-based films can be used as packaging materials (16). Plasticizers can increase the EAB and reduce the TS of starch films because their addition leads to changes in the network structure of starch films (28). When a plasticizer is added to starch films, the starch structure becomes less dense, and the movement of the polymer chain is promoted under tension (29). As shown in Table 1 and Figure 2, the TS of the corn starch-based films decreased with the increases in the GLY ratio. The TS decreased from 18.86 ± 0.30 MPa to 4.35 ± 0.16 MPa, and the EAB increased from 12.52 ± 2.11% to 73.38 ± 2.22%. TS may have increased due to the difference in plasticization between GLY and ERY. The molecular weight of GLY is lower than that of ERY (GLY 92 g/mol, ERY 122 g/mol), so GLY can more easily enter the molecular chain of starch to increase the molecular space between polymer chains than ERY, thus reducing the number of hydrogen bonds in the starch chain (25). The EAB of the corn starch-based films increased with increasing GLY ratio. The plasticizer molecules enter the starch matrix and occupy the space through hydrogen bonds, destroying the structure of the polymer; thus, the starch structure is transformed into a flexible mesoporous structure with greater fluidity (30–32). The molecular chain of GLY is smaller than that of ERY, so GLY can enter the polymer network more easily than ERY.

### Water Vapor Permeability

The primary function of food packaging materials is to avoid contact between food and external media and control the water exchange between food and the environment; therefore, WVP is an important parameter of packaging materials (33). As shown in Table 2, the WVP of corn starch-based films increased with the increases in the GLY ratio. With an increase in the plasticizer content in the biopolymer, the molecular structure of the polymer changes, the intermolecular force decreases, the free volume of the system increases, and the network structure becomes loose so that water molecules can pass through starch films (34, 35). Compared with ERY, GLY has a smaller molecular weight, so it is easier to enter into corn starch granules, playing a plasticizing role. With the increase of ERY ratio, the plasticizing effect of corn starch-based corn starch films weakened. The degree of damage to the molecular structure is not as substantial as that of glycerol. So the damage degree of the molecular structure decreases gradually with the increase of the proportion of ERY. It is hard for water molecules to pass through the corn starch-based films. Hence, the WVP of the corn starch-based films decreases with the increase of the proportion of ERY. Ballesteros-Mártinez et al. (15) concluded that the WVP of starch films increases with an increase in the GLY ratio.

**TABLE 2:** WVP and OP of the corn starch-based films supplemented with GLY and ERY (4:0, 3:1, 2:2, 1:3, 0:4).

Sample	Water vapor permeability (WVP) (g mm^–2^⋅s^–1^Pa^–1^)	Oxygen permeability (OP) (cm^2^s^–1^Pa^–1^)
GLY: ERY (4:0)	3.44 ± 0.03^a^	10.82 ± 0.68^a^
GLY: ERY (3:1)	3.33 ± 0.07^a^	8.31 ± 0.78^b^
GLY: ERY (2:2)	2.38 ± 0.05^b^	6.24 ± 1.32^c^
GLY: ERY (1:3)	2.37 ± 0.02^b^	5.01 ± 0.25^d^
GLY: ERY (0:4)	2.27 ± 0.03^b^	2.68 ± 0.12^e^

*Different superscript letters in each column showed a significant difference (P < 0.05).*

### Oxygen Permeability

Oxygen permeability is an important indicator of packaging materials. A lower OP can reduce the contact angle between oxygen and the internal environment of the packaging. As shown in Table 2, an increase of GLY ratio increased the OP of corn starch-based films (from 10.82 ± 0.68 to 2.68 ± 0.12 cm^2^s^–1^Pa^–1^), indicating that GLY had a significant effect on the OP of corn starch-based films. The reason behind that could be attributed to the molecular weight of GLY, which is smaller than that of ERY, making it easier for GLY to enter starch granules and destroy the intermolecular bonds of starch, thus softening the internal bulk structure of corn starch-based films. As a result, corn starch-based films’ internal oxygen transport channel increases. In turn, the OP of corn starch-based films increases with the increase of GLC concentration. Siripatrawan and Vitchayakitti (36) reported that the microstructure, pore volume, arrangement of polymer chains, and the membrane matrix’s binding force greatly influence the OP of starch films.

### Water Solubility

The water solubility of starch films is an important index of packaging materials and has a wide range of applications (14). Some packages require starch films with low water solubility to maintain the integrity of the packaging materials. In contrast, some starch films may need high water solubility for freshly processed products (15). With the increase of GLY ratio, the water solubility of corn starch-based films increased (from 25.04 ± 1.81% to 36.56 ± 3.58%) with significant differences among groups (*P* < 0.05) (Table 1). It has been reported that the addition of plasticizers to starch films can reduce the interaction between biological polymer chains and enhance the interaction between the plasticizer and polymer, thus increasing the water solubility of starch films. The addition of a plasticizer modifies the polymer molecular network, and the hydrogen bond interferes with the polymer network to reduce the density of interactions between starch polymer molecules. It increases the water solubility of starch films (34, 35, 37). As shown in Table 1, the water solubility of corn starch-based films plasticized by GLY was higher than that of corn starch-based films plasticized by ERY. The reason is that the molecular weight of GLY is lower than that of ERY and because it is easier for GLY to insert between polymer chains, the ability of GLY to interfere with starch network interaction is stronger (15).

### Water Contact Angle

**FIGURE 3:**
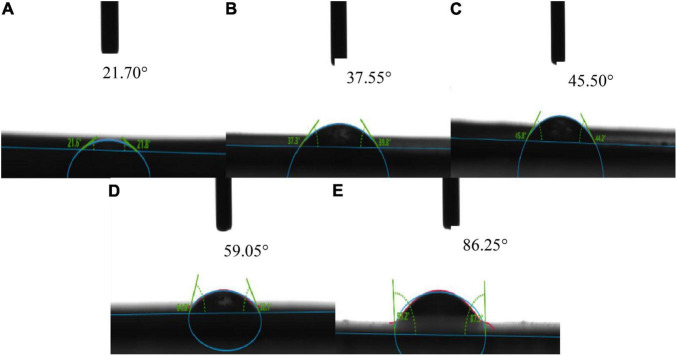
The water contact angle of corn starch-based films supplemented with GLY and ERY **(A)** (0:4), **(B)** (1:3), **(C)** (2:1), **(D)** (3:1), and **(E)** (4:0).

The water contact angle is an important index for determining the efficiency of packaging materials (38). The size of the water contact angle can determine whether the packaging material is hydrophilic or hydrophobic (39). When the water contact angle is greater than 90°, the packaging material is hydrophobic, and when the contact angle is less than 90°, the packaging material is hydrophilic. As shown in Figure 3, the contact angle of corn starch-based films was always less than 90°, indicating that corn starch-based films were hydrophilic independent of the GLY to ERY ratio. When the GLY: ERY was 0:4, the water contact angle of the corn starch-based films were the largest, indicating that the hydrophilicity of corn starch-based films was the lowest. This observation could be that ERY is not hydrophilic, so it has a large contact angle when the ratio of GLY: ERY is 4:0 (40). With the increase in GLY ratio, the contact angle of corn starch-based films decreased, indicating that the hydrophilicity was enormously increased. It was reported that the addition of GLY to starch films increases the hygroscopicity of the starch films (41). Wang et al. (6) also showed that the addition of GLY increases the hydrophilicity of starch films.

### X-Ray Diffraction

**FIGURE 4:**
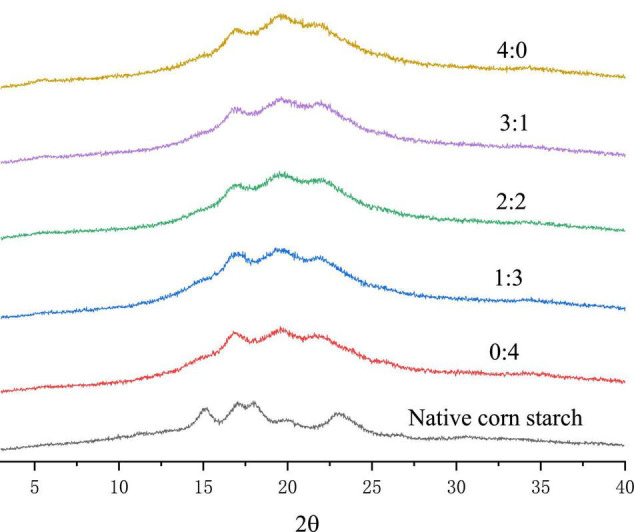
XRD of corn starch-based films supplemented with GLY and ERY (4:0, 3:1, 2:2, 1:3, and 0:4).

The crystallinity of corn starch-based films with different GLY to ERY ratios is shown in Figure 4. Corn starch belongs to the type A crystallization group, and the diffraction peaks are at 15°, 17°, 18°, and 23° (42). After gelatinization, corn starch has a mixed crystallization peak of B-type and V-type (43, 44). Our results showed that the gelatinization of corn starch caused the A-type crystallization of corn starch to be replaced by B- and V-type crystallization. With an increase in GLY ratio, the intensity of the V-type crystal diffraction peak at 19.8° increased, indicating that the addition of GLY increased the V-type crystallization of corn starch-based films. In this context, Liu et al. (45) reported two reasons for forming V-type crystallization in starch films. First, the lipid in starch forms a lipid complex with amylose, and second, the addition of GLY can promote the increase of V-type crystallization in corn starch-based films. Further, Zou et al. (46) also concluded that GLY promotes the V-shaped crystallization of corn starch-based films.

### Thermal Stability

**FIGURE 5:**
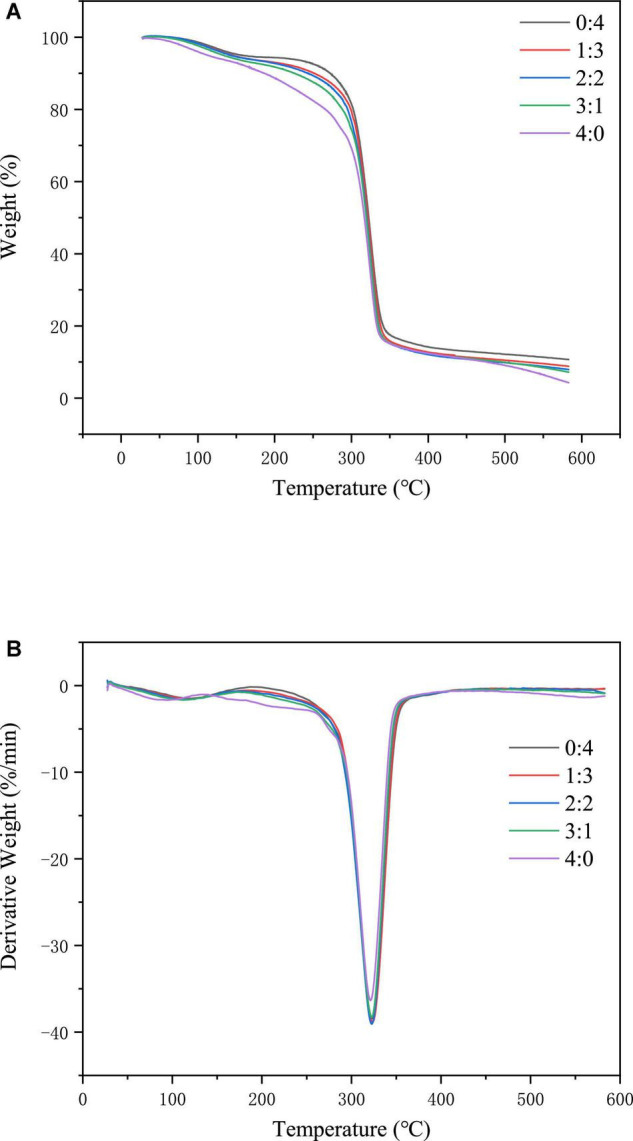
TGA **(A)** and DTG **(B)** of corn starch-based films supplemented with GLY and ERY (4:0, 3:1, 2:2, 1:3, and 0:4).

As shown in Figure 5A, the weight loss of the corn starch-based films consisted of three stages. The weight loss in the first stage occurred between 30 and 130°C. The main reason for the weight loss at this stage was the loss of water in the corn starch-based films. The second weight-loss stage occurred between 250 and 400°C; the leading cause was starch degradation. The third stage of weight loss occurred between 400 and 600°C, and the main cause was the reduction of ash.

It can be implied from Figure 5B that the maximum degradation temperature of corn starch-based films decreased gradually with the increase of GLY ratio (from 326.84 to 324.36°C), denoting that the increase in GLY ratio diminished the thermal stability of corn starch-based films. The plasticization mechanism of GLY involves the binding through the hydroxyl groups of GLY and those of starch, which leads to a reduction in the binding of hydroxyl groups between starch molecules. The force between starch molecules is destroyed, resulting in a decrease in the thermal stability of the corn starch-based films. On this occasion, Gao et al. (41) concluded that the maximum degradation temperature of starch films decreases with an increase in the ratio of GLY and water. This finding was similar to the results of our experiment.

### AFM

Figure 5 shows three-dimensional images of corn starch-based films plasticized with different ratios of GLY and ERY. In addition, the roughness parameters (Rq, Ra) of corn starch-based films can be calculated according to AFM images. As shown in Table 3, the roughness of corn starch-based films decreases with the increase of the GLY ratio (Rq: 403–260 nm). The reason could be attributed to the fact that the plasticizing effect of ERY is less than that of GLY, which is easy to cause the phase separation between starch and ERY. As a result, the surface roughness of corn starch-based films increases with the decrease of the ratio of GLY. In this context, Nguyen Vu and Lumdubwong (47) revealed that when sorbitol and GLY are used to plasticize cassava starch films, the roughness of cassava starch films increases gradually with the increase of sorbitol ratio. In addition, ERY has high crystallinity, which would increase the surface roughness of corn starch-based films. As stated elsewhere, the film would have a rough appearance due to the high crystallinity (48).

**TABLE 3:** AFM of the corn starch-based films supplemented with GLY and ERY (4:0, 3:1, 2:2, 1:3, 0:4).

Sample	Rq (nm)	Ra (nm)
GLY: ERY (4:0)	260	209
GLY: ERY (3:1)	273	228
GLY: ERY (2:2)	279	230
GLY: ERY (1:3)	284	245
GLY: ERY (0:4)	403	315

## Conclusion

Herein, the physicochemical properties of corn starch-based films with different GLY/ERY ratios were investigated. With the increase of the GLY ratio, the barrier performance (WVP and OP) of corn starch-based films increased. In addition, the mechanical properties of corn starch-based films changed significantly with the increase of the GLY ratio (TS decreased and EAB increased). The basic physicochemical properties (thickness, water solubility, and moisture content) of corn starch-based films decreased with the increase of GLY ratio. Further, GLY has a greater effect on the thermal stability of corn starch-based films than ERY. The experimental results can provide a theoretical basis for the production of high barrier packaging materials, such as tea packaging.

## Data Availability Statement

The original contributions presented in the study are included in the article/supplementary material, further inquiries can be directed to the corresponding authors.

## Author Contributions

BW: investigation, software, visualization, and writing – original draft. XX and YF: supervision and project administration. SY: formal analysis. BC and AMA: conceptualization, methodology, writing–review and editing, and supervision. All authors contributed to the article and approved the submitted version.

## Conflict of Interest

The authors declare that the research was conducted in the absence of any commercial or financial relationships that could be construed as a potential conflict of interest.

## Publisher’s Note

All claims expressed in this article are solely those of the authors and do not necessarily represent those of their affiliated organizations, or those of the publisher, the editors and the reviewers. Any product that may be evaluated in this article, or claim that may be made by its manufacturer, is not guaranteed or endorsed by the publisher.

## References

[1] Alaaeddin MH Sapuan SM Zuhri MYM Zainudin ES Al-Oqla FM. Photovoltaic applications: status and manufacturing prospects. *Renew Sustain Energy Rev.* (2019) 102:318–32. 10.1016/j.rser.2018.12.026

[2] Halley PJ Dorgan JR. Next-generation biopolymers: advanced functionality and improved sustainability. *MRS Bull.* (2011) 36:687–91. 10.1186/s12934-020-01456-4 33183275 PMC7659065

[3] Qi RM Jones DL Li Z Liu Q Yan CR. Behavior of microplastics and plastic film residues in the soil environment: a critical review. *Sci Total Environ.* (2020) 703:134722. 10.1016/j.scitotenv.2019.134722 31767311

[4] Hirsch RL. Mitigation of maximum world oil production: shortage scenarios. *Energy Policy.* (2008) 36:881–9. 10.1016/j.enpol.2007.11.009

[5] Benhamou K Dufresne A Magnin A Mortha G Kaddami H. Control of size and viscoelastic properties of nanofibrillated cellulose from palm tree by varying the TEMPO-mediated oxidation time. *Carbohydr Polym.* (2014) 99:74–83. 10.1016/j.carbpol.2013.08.032 24274481

[6] Wang B Yu B Yuan C Guo L Liu P Gao W An overview on plasticized biodegradable corn starch-based films: the physicochemical properties and gelatinization process. *Crit Rev Food Sci Nutr.* (2021). [Online ahead of print] 10.1080/10408398.2020.1868971 33401939

[7] do Val Siqueira L Arias CILF Maniglia BC Tadini CC. Starch-based biodegradable plastics: methods of production, challenges and future perspectives. *Curr Opin Food Sci.* (2021) 38:122–30. 10.1016/j.cofs.2020.10.020

[8] Cioica N Fechete R Cota C Nagy EM David L Cozar O. NMR relaxation investigation of the native corn starch structure with plasticizers. *J Mol Struct.* (2013) 1044:128–33. 10.1016/j.molstruc.2013.01.037

[9] Pérez S Bertoft E. The molecular structures of starch components and their contribution to the architecture of starch granules: a comprehensive review. *Starch.* (2010) 62:389–420. 10.1002/star.201000013

[10] Talja RA *Preparation and Characterization of Potato Starch Films Plasticized with Polyols*. Academic dissertation. Helsinki: Faculty of Agriculture and Forestry of the University of Helsinki (2007).

[11] Goossens J Gonze M. Erythritol. *Manuf. Confectioner* (2000) 80:71–5.

[12] Talja RA Helén H Roos YH Jouppila K Effect of various polyols and polyol contents on physical properties of potato starch-based films. *Carbohydr. Polym*. (2007) 67:288–95.

[13] Fernández Cervera M Karjalainen M Airaksinen S Rantanen J Krogars K Heinämäki J Physical stability and moisture sorption of aqueous chitosan-amylose starch films plasticized with polyols. *Eur. J. Pharm. Biopharm*. (2004) 58:69–76. 15207539 10.1016/j.ejpb.2004.03.015

[14] Wang B Yan S Gao W Kang X Yu B Liu P Antibacterial activity, optical, and functional properties of corn starch-based films impregnated with bamboo leaf volatile oil. *Food Chem.* (2021) 357:129743. 10.1016/j.foodchem.2021.129743 33866242

[15] Ballesteros-Mártinez L Pérez-Cervera C Andrade-Pizarro R. Effect of glycerol and sorbitol concentrations on mechanical, optical, and barrier properties of sweet potato starch film. *NFS J.* (2020) 20:1–9. 10.1016/j.nfs.2020.06.002

[16] Wang B Sui J Yu B Yuan C Guo L Abd El-Aty AM Physicochemical properties and antibacterial activity of corn starch-based films incorporated with *Zanthoxylum bungeanum* essential oil. *Carbohydr Polym.* (2021) 254:117314. 10.1016/j.carbpol.2020.117314 33357877

[17] Suh JH Ock SY Park GD Lee MH Park HJ. Effect of moisture content on the heat-sealing property of starch films from different botanical sources. *Polym Testing.* (2020) 89:106612. 10.1016/j.polymertesting.2020.106612

[18] Wang B Gao W Kang X Dong Y Liu P Yan S Structural changes in corn starch granules treated at different temperatures. *Food Hydrocoll.* (2021) 118:106760. 10.1016/j.foodhyd.2021.106760

[19] Tian Y Li Y Xu X Jin Z. Starch retrogradation studied by thermogravimetric analysis (TGA). *Carbohydr Polym.* (2011) 84:1165–8. 10.1016/j.carbpol.2011.01.006

[20] Ruggero F Carretti E Gori R Lotti T Lubello C. Monitoring of degradation of starch-based biopolymer film under different composting conditions, using TGA, FTIR and SEM analysis. *Chemosphere.* (2020) 246:125770. 10.1016/j.chemosphere.2019.125770 31901665

[21] Ghasemlou M Aliheidari N Fahmi R Shojaee-Aliabadi S Keshavarz B Cran MJ Physical, mechanical and barrier properties of corn starch films incorporated with plant essential oils. *Carbohydr Polym.* (2013) 98:1117–26. 10.1016/j.carbpol.2013.07.026 23987453

[22] Edhirej A Sapuan SM Jawaid M Zahari NI. Effect of various plasticizers and concentration on the physical, thermal, mechanical, and structural properties of cassava-starch-based films. *Starch.* (2017) 69:1500366. 10.1002/star.201500366

[23] Nordin N Othman SH Rashid SA Basha RK. Effects of glycerol and thymol on physical, mechanical, and thermal properties of corn starch films. *Food Hydrocoll.* (2020) 106:105884. 10.1016/j.foodhyd.2020.105884

[24] Lim WS Ock SY Park GD I Lee W Lee MH Park HJ. Heat-sealing property of cassava starch film plasticized with glycerol and sorbitol. *Food Packag Shelf Life.* (2020) 26:100556. 10.1016/j.fpsl.2020.100556

[25] Orsuwan A Sothornvit R. Effect of banana and plasticizer types on mechanical, water barrier, and heat sealability of plasticized banana-based films. *J Food Process Preserv.* (2018) 42:e13380. 10.1111/jfpp.13380

[26] Galdeano MC Mali S Grossmann MVE Yamashita F García MA. Effects of plasticizers on the properties of oat starch films. *Mater Sci Eng C.* (2009) 29:532–8. 10.1016/j.msec.2008.09.034

[27] Sanyang ML Sapuan SM Jawaid M Ishak MR Sahari J. Effect of plasticizer type and concentration on physical properties of biodegradable films based on sugar palm (*Arenga pinnata*) starch for food packaging. *J Food Sci Technol.* (2016) 53:326–36. 10.1007/s13197-015-2009-7 26787952 PMC4711441

[28] Farahnaky A Saberi B Majzoobi M. Effect of glycerol on physical and mechanical properties of wheat starch edible films. *J Texture Stud.* (2013) 44:176–86. 10.3390/polym10040412 30966447 PMC6415220

[29] Dias AB Müller CMO Larotonda FDS Laurindo JB. Biodegradable films based on rice starch and rice flour. *J Cereal Sci.* (2010) 51:213–9. 10.1016/j.jcs.2009.11.014

[30] Atarés L De Jesús C Talens P Chiralt A. Characterization of SPI-based edible films incorporated with cinnamon or ginger essential oils. *J Food Eng.* (2010) 99:384–91. 10.1016/j.jfoodeng.2010.03.004

[31] Ghasemlou M Khodaiyan F Oromiehie A. Physical, mechanical, barrier, and thermal properties of polyol-plasticized biodegradable edible film made from kefiran. *Carbohydr Polym.* (2011) 84:477–83. 10.1016/j.carbpol.2010.12.010

[32] Razavi SMA Mohammad Amini A Zahedi Y. Characterisation of a new biodegradable edible film based on sage seed gum: influence of plasticiser type and concentration. *Food Hydrocoll.* (2015) 43:290–8. 10.1016/j.foodhyd.2014.05.028

[33] Hosseini SF Rezaei M Zandi M Ghavi FF. Preparation and functional properties of fish gelatin–chitosan blend edible films. *Food Chem.* (2013) 136:1490–5. 10.1016/j.foodchem.2012.09.081 23194553

[34] Sothornvit R Krochta JM. Plasticizer effect on oxygen permeability of β-Lactoglobulin films. *J Agric Food Chem.* (2000) 48:6298–302. 10.1021/jf000836l 11312802

[35] Nogueira GF Fakhouri FM de Oliveira RA. Extraction and characterization of arrowroot (Maranta arundinaceae L.) starch and its application in edible films. *Carbohydr Polym.* (2018) 186:64–72. 10.1016/j.carbpol.2018.01.024 29456010

[36] Siripatrawan U Vitchayakitti W. Improving functional properties of chitosan films as active food packaging by incorporating with propolis. *Food Hydrocoll.* (2016) 61:695–702. 10.3390/foods10051110 34067772 PMC8156044

[37] Saberi B Thakur R Vuong QV Chockchaisawasdee S Golding JB Scarlett CJ Optimization of physical and optical properties of biodegradable edible films based on pea starch and guar gum. *Ind Crops Prod.* (2016) 86:342–52. 10.1016/j.indcrop.2016.04.015

[38] Zhang D Chen L Cai J Dong Q Din Z-U Hu Z-Z Starch/tea polyphenols nanofibrous films for food packaging application: from facile construction to enhance mechanical, antioxidant and hydrophobic properties. *Food Chem.* (2021) 360:129922. 10.1016/j.foodchem.2021.129922 33965711

[39] Fan J-B Song Y Wang S Meng J Yang G Guo X Directly coating hydrogel on filter paper for effective oil–water separation in highly acidic, alkaline, and salty environment. *Adv Funct Mater.* (2015) 25:5368–75. 10.1002/adfm.201501066

[40] Fry JC. Natural low-calorie sweeteners. In: Baines D Seal R *Natural Food Additives, Ingredients and Flavourings.* Sawston: Woodhead Publishing (2012). p. 41–75. 10.1533/9780857095725.1.41

[41] Gao W Zhu J Kang X Wang B Liu P Cui B Development and characterization of starch films prepared by extrusion blowing: the synergistic plasticizing effect of water and glycerol. *LWT Food Sci Technol.* (2021) 148:111820. 10.1016/j.lwt.2021.111820

[42] Bertoft E. Lintnerization of two amylose-free starches of A- and B-crystalline types, respectively. *Starch.* (2004) 56:167–80. 10.1002/star.200300255

[43] Zhang B Chen L Zhao Y Li X. Structure and enzymatic resistivity of debranched high temperature–pressure treated high-amylose corn starch. *J Cereal Sci.* (2013) 57:348–55. 10.1016/j.jcs.2012.12.006

[44] Dang X Chen H Wang Y Shan Z. Freeze-drying of oxidized corn starch: electrochemical synthesis and characterization. *Cellulose.* (2018) 25:2235–47. 10.1007/s10570-018-1701-y

[45] Liu H Adhikari R Guo Q Adhikari B. Preparation and characterization of glycerol plasticized (high-amylose) starch–chitosan films. *J Food Eng.* (2013) 116:588–97. 10.1016/j.jfoodeng.2012.12.037

[46] Zou Y Yuan C Cui B Liu P Wu Z Zhao H. Formation of high amylose corn starch/konjac glucomannan composite film with improved mechanical and barrier properties. *Carbohydr Polym.* (2021) 251:117039. 10.1016/j.carbpol.2020.117039 33142597

[47] Nguyen Vu HP Lumdubwong N. Starch behaviors and mechanical properties of starch blend films with different plasticizers. *Carbohydr Polym.* (2016) 154:112–20. 10.1016/j.carbpol.2016.08.034 27577902

[48] Araújo A Galvão A Filho CS Mendes F Oliveira M Barbosa F Okra mucilage and corn starch bio-based film to be applied in food. *Polymer Testing.* (2018) 71:352–61. 10.1016/j.polymertesting.2018.09.010

